# Tennis elbow, study protocol for a randomized clinical trial: needling with and without platelet-rich plasma after failure of up-to-date rehabilitation

**DOI:** 10.1186/s13018-020-01998-8

**Published:** 2020-10-07

**Authors:** A. J. Schwitzguebel, M. Bogoev, V. Nikolov, F. Ichane, A. Lädermann

**Affiliations:** 1 Sports Medicine Division, La Providence Hospital, Fbg de l’Hopital 81, 2000 Neuchâtel, Switzerland; 2 Giant Studio, Rue des Noyers 2, 2000 Neuchâtel, Switzerland; 3 Service Médecine interne, Groupe Hospitalier Est Reunion, 30 rte Nationale 3 - ZAC Madeleine, 186 97470 Saint Benoît, BP France; 4 Division of Orthopaedics and Trauma Surgery, La Tour Hospital, Rue J.-D. Maillard 3, 1217 Meyrin, Switzerland; 5 Faculty of Medicine, University of Geneva, Rue Michel-Servet 1, 1211 Geneva 4, Switzerland; 6 Division of Orthopaedics and Trauma Surgery, Department of Surgery, Geneva University Hospitals, Rue Gabrielle-Perret-Gentil 4, CH-1211, 14 Geneva, Switzerland

**Keywords:** Clinical protocols, Double-blind method, Randomized, Research design, Tennis Elbow, Tendinopathy, Rehabilitation, Extracorporeal shock waves therapy, Platelet-rich plasma, Ultrasonography, Interventional

## Abstract

**Background:**

The conservative management of lateral epicondylitis is known to be a difficult-to-treat annoying condition. A treatment with platelet-rich plasma (PRP) is often performed, but its efficacy remains controversial.

**Methods:**

This study is a single-center, randomized double-blind controlled trial, preceded by a case series. All the 232 planned patients of the case series will undergo an up-to-date comprehensive rehabilitation program, including focused extracorporeal shock waves therapy. This rehabilitation program is expected to have a maximum success rate 75%. It is therefore aimed to allocate a minimum of 58 patients with rehabilitation failure into the 1:1 randomized trial. Stratification is planned on age and lesion pattern. The masking will be quadruple (Participant, Care Provider, Investigator & Outcome Assessor). The patients will undergo an ultrasound (US)-guided needling combined with either PRP (intervention group) or saline (control group). The primary endpoint will be the pain improvement from baseline (month 0) at 3 months on a 0–10 visual analog scale (VAS) during a maximal strength isometric contraction of the extensor carpialis brevis muscle. The main secondary endpoints will include the rehabilitation success rate and improvements from baseline at 3, 6, and 12 months of the following outcomes: (i) Single Assessment Numeric Evaluation (SANE) score, (ii) Patient-Rated Tennis Elbow Evaluation (PRTEE) score, (iii) maximal grip strength on Jamar test, and (iv) the ultrasonographic evaluation of the US of the epicondylar tendons.

**Discussion:**

The study results will provide insight into the effect of PRP as adjuvant therapy to tendon fenestration, and may contribute to identify the best preceding and concomitant rehabilitation protocol.

**Trial registration:**

ClinicalTrials.gov NCT03987256. Registered on 20 August 2019.

## Background

The conservative management of lateral epicondylitis is known to be a difficult-to-treat annoying condition. The first-line conservative management includes physical therapies, orthotics [1], and extracorporeal shock wave therapy (ESWT) [2]. The success rate of ESWT for lateral epicondylitis depends mainly on the protocol that is followed. For instance, poor results have been observed with too low energy [3]. Focused ESWT has been showed to be as effective as surgical tenotomy [4].

Infiltrative therapies might be proposed in case of persistent symptoms. It has been well established that corticosteroids are efficient in the short-term but deleterious in the long-term [5, 6] likely for degenerative purposes [7]. Prolotherapy, autologous blood or botulinic toxin injections, and others infiltrative therapies are less studied and are therefore not clearly supported by the current literature [8–10]. Stem cells might be an appropriate alternative in the future [11].

Platelet-rich-plasma (PRP) is nowadays widely used for tendinopathies, considered as safe, and currently supported by the strongest scientific journals [12]. However, the potential benefits of PRP are discordant, especially concerning the elbow. Even if the superiority of PRP over corticosteroids is well established [13], the efficacy of PRP in addition to tendon needling or fenestration compared to tendon needling or fenestration alone is still controversial [14–19].

Several factors have been advocated to influence PRP outcomes. The most relevant ones are direct mechanical action of the needle and fenestration technique, number of PRP injections, cell counts (platelets, white blood and red blood cells), activation of the platelets, concomitant local anesthetic use, peri-interventional use of NSAIDs and corticosteroids, concomitant rehabilitation, or a contrario immobilization [20]. The positive results observed in the previous reported studies remain debatable as they can be [17, 18] related to either PRP, fenestration [21], or any of the abovementioned confounding factors.

The first aim of this study is to determine the proportion of patients that would need an infiltrative technique after a proper rehabilitation protocol including physical therapies, focused ESWT, and orthotics and Kinesio taping for all patients. Our second aim is to establish whether PRP as adjuvant therapy to fenestration would increase clinical outcomes.

## Methods

### Aims

1. The clinical efficacy of platelet-rich plasma as adjuvant therapy to tendon needling for patients suffering of epicondylar tendinosis managed with a first line up-to-date rehabilitation
2. The efficacy of platelet-rich plasma as adjuvant therapy to tendon needling on the epicondylar tendon repair
3. The clinical efficacy of the first-line rehabilitation
4. The efficacy of the first-line rehabilitation on the epicondylar tendon repair

### Study design

**Fig. 1:**
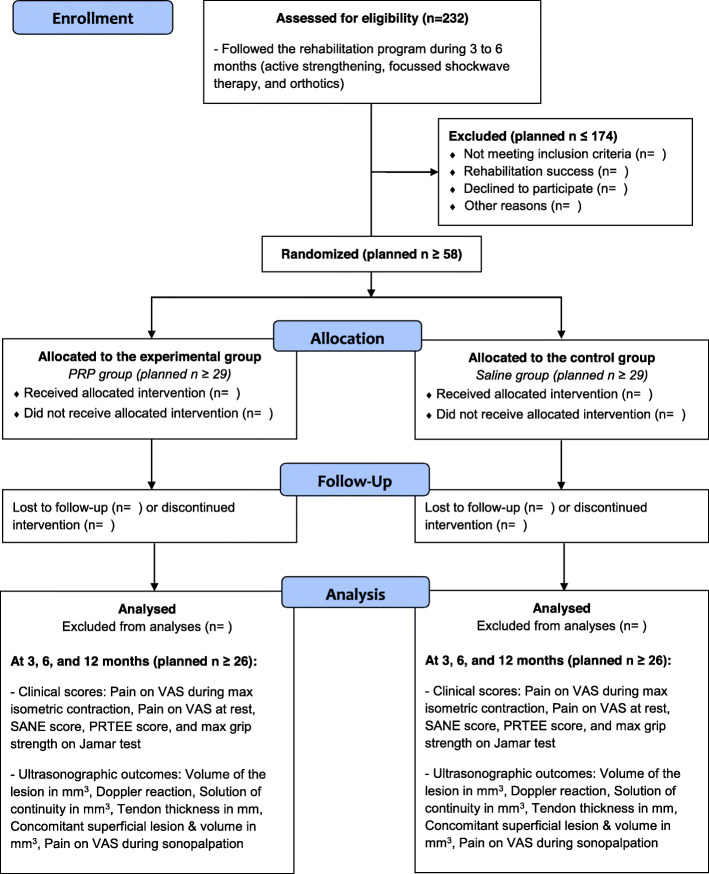
Study flowchart

This study will include 232 patients and will be conducted in two steps (Fig. 1). The first step consists in an observational case series. During this step, all patients will benefit from a proper rehabilitation including epicondylar stretching and eccentric strengthening; periscapular and global tonification; postural adjustment; manual therapies including trigger points release, epicondylar taping, or bracing; and focused shockwave therapy (Additional file 1). The second step consists in a case-control superiority trial randomized 1:1 between PRP (intervention group) and saline (control group) injections. A stratification is planned on age and lesion pattern.

Timelines are presented in Table 1. After 3 to 6 months of rehabilitation, if patients are not satisfied, they will be allocated to the PRP or saline group. The follow-up will last 1 year after the intervention (intervention is at month 0), with endpoints at − 3/− 6, 0, 3, 6, and 12 months of follow-up.

**Table 1:** Participant timeline

		Study period								
	Enrolment	Rehabilitation				Allocation	Post-allocation			Close out
**Timepoint in months**	− 3^¼^ to − 6^¼^	− 3 to − 6	− 1^½^ to − 4^½^	0 to − 3	0*	0	¼	3	6	12
**Enrolment**										
Patient information and consent	x									
Informed consent collection		x								
Validation of inclusion and exclusion criteria		x								
Randomization						x				
**Interventions**										
Organize rehabilitation		x								
Rehabilitation checkpoint			x	x	x			x	x	
Needling (with PRP or Saline)							x			
**Assessment**										
Clinical evaluation	x	x								
Elbow echography	x			x	x			x	x	
Demographics		x								
Rehabilitation efficacy assessment				x	x					
Primary outcome assessment								x		
Clinical outcome assessment		x		x	x		x	x	x	x
Concomitant therapy, surgery			x	x	x		x	x	x	x

*Extra rehabilitation visit if rehabilitation lasts > 3 months

### Participants

In this monocentric study, all patients will be recruited into the sports medicine division of La Providence Hospital, Neuchâtel, Switzerland. The following inclusion criteria will be applied: (i) lesion of the extensor carpalis radialis brevis (ECRB) tendon on ultrasonography; (ii) age between 18 and 65 years old; and (iii) written consent obtained following adequate explanation of the study aims, design, and procedures. The following exclusion criteria will be applied: (i) presence of a concomitant pathology that can partially explain the symptoms; (ii) diabetes, immunocompromised status, bleeding disorder, or other significant systemic disease; (iii) corticoids or anticoagulants intake; (iv) anticoagulation therapy; and (v) allergy to local anesthetics.

The informed consent will be obtained by the sponsor-investigator (AS). Other physicians in La Providence Hospital and connected structures in Neuchâtel city (i.e., orthopedists, general practitioners, physiotherapists) are informed of the project. They are kindly requested to refer patients to the sports medicine department.

### Randomization and blinding

The allocation will be performed using computer-generated random number by serials of 4 patients blocks on a 1:1 ratio. Four strata will be used: (i) age 18–39 and absence of lesion on the superficial epicondylar tendon; (ii) age 18–39 and concomitant lesion on the superficial epicondylar tendon; (iii) age 40–65 and absence of lesion on the superficial epicondylar tendon; and (iv) age 40–65 and concomitant lesion on the superficial epicondylar tendon. The sponsor-investigator (AS) will generate 10 computerized allocation sequences. The secretary will randomly select one of those sequences for the study. The sponsor-investigator will enroll the participants and will perform the intervention 3 months after the recruitment. Patients will be allocated to a group at the time of the intervention, and only if the rehabilitation process was not successful. The unblinded co-investigator (MF) will access to the allocation on a sequentially numbered list and prepare the injection for the intervention in an opaque syringe (blinded content for AS). It is worth noting that this co-investigator will not take care of the patients subsequently.

This study is quadruple-blinded. Blinding concerns (i) the participants and (ii) the principal investigator (AS) during the intervention, (iii) the outcome assessment, and (iv) the statistical analysis. A complete masking of the intervention will allow the blinding of the participant and the principal investigator for the intervention and the outcome assessment. First, for all patients, the co-investigator (MF) will perform a blood puncture in room A. He will then go in room B and, depending of the allocation, prepare the PRP or turn-on the centrifuge for 5 min (sham PRP preparation). After that, he will prepare an opaque syringe containing either PRP or saline solution. In room A, the principal investigator will perform the ultrasound-guided fenestration of the ECRB tendon. During the fenestration, he will use the opaque syringe and inject the blinded content into the ECRB tendon. The statistical analysis (iv) will be performed by the principal investigator using a sham allocation list. Once the statistical analysis is completed, the true allocation list will replace the former list.

Unblinding should not be necessary for the security of patients. Indeed, no adverse effects of the PRP (local pain and tenderness, infection) require unblinding for clinical management. Despite this, if unblinding is necessary in case of unexpected circumstance, the co-investigator will reveal the intervention for concerned patients.

### Study treatments and interventions

All patients should perform the rehabilitation protocol during the 3 months following the intervention (Additional file 1). During this protocol, all patients will perform global and specific active strengthening and self-stretching, focused ESWT, focused therapies, Kinesio taping, and orthotics. After 3 months, if a constant but insufficient symptom alleviation is observed, the rehabilitation program could be lengthened by 3 months (i.e., rehabilitation months 4–6). If a radiological ECRB lesion persists on ultrasonography, shockwave therapy will be performed during rehabilitation months 4–6. If the rehabilitation protocol enables symptom disappearance (i.e., the rehabilitation was successful), the intervention will not be performed. If, for any other reason, the intervention cannot be performed, the patient will be dropped out of the study.

It is not clear whether PRP should be used or not as an adjuvant procedure to tendon needling. An injection of saline solution during the procedure as comparator seems the most logical, because it (i) allows a proper blinding of both patient and physician, (ii) has no deleterious effects on tendon repair (contrary to active products such as corticosteroids) and therefore no influence on clinical outcomes, and (iii) has no potential side effects.

In both groups, the intervention will occur under strict aseptic conditions as following: First, a local anesthetic blockade of the radial nerve under the arcade of Frohse will be performed under ultrasonographic control. Then, an ultrasound-guided needling of the ECRB tendon will be performed, using the fenestration technique: 25 repetitions with a 20-gauge needle. During the procedure, the lesion will be fulfilled with either PRP (experimental group) or saline solution (control group). The PRP (ACP Arthrex) will be prepared using a double syringe system under the manufacturer’s recommendation. From 15 ml of peripheral blood, the PRP will be extracted on site using centrifugation (1500 rpm 5 min), as recommended by the manufacturer. After the intervention, the learned self-exercises should be continued as long as necessary. The use of ice and pain medication with paracetamol or opioids (tramadol, codeine) will be used to manage pain, if necessary.

No particular measures are planned to improve the adherence to the intervention. The adherence to the rehabilitation protocol will be optimized by the physician, because (i) he will encourage the patient and actively participate to the rehabilitation protocol (Additional file 1) during shockwaves sessions and (ii) at 6 weeks of rehabilitation, the patient will be asked to show the learned active auto-exercises to the physician. Oral corticosteroids, aspirin, and non-steroid anti-inflammatory drugs must be avoided, in spite of their potential deleterious effect on focused shockwaves and PRP. If patients remain symptomatic at the end of the trial, a second needling of the tendon with PRP as adjuvant therapy might be considered. If relevant, a surgical debridement of the lesion will be considered.

### Outcome measurements and assessments

The primary endpoint is the pain improvement between months 0 and 3 on a 0–10 visual analog scale (VAS) during a maximal strength isometric contraction of the extensor carpialis brevis muscle. Rationale: most specific clinical test in order to reproduce pain triggered by the tendon target of the intervention. Most clinical effects of the PRP use are awaited after 3 months of follow-up [18, 22]. The tendon healing will be evaluated with ultrasound in secondary outcomes. It was decided to use clinical parameters as primary outcome, because some pathological elements might persist on ultrasound after healing. Although the sensitivity of ultrasound is high, it does not reach 100% [23, 24].

Secondary endpoints:

Proportion of patients for which the tendon needling is not necessary after rehabilitation protocol

Clinical scores at − 3, 0, 3, 6, and 12 months of follow-up: (i) pain on a 0–10 (VAS) during a maximal strength isometric contraction at other timepoints; (ii) pain on a 0–10 VAS scale at rest; (iii) Single Assessment Numeric Evaluation (SANE) score; (iv) Patient-Rated Tennis Elbow Evaluation (PRTEE) score; and (v) maximal grip strength on Jamar test.

Ultrasonographic aspects of the epicondylar tendons at − 3, 0, 3, 6, and 12 months of follow-up: (i) volume of the lesion in mm^3^; (ii) Doppler reaction classified at the proportion of the tendon marked with the Doppler signal; (iii) solution of continuity in mm^3^; (iv) tendon thickness in mm; (v) concomitant superficial lesion and volume in mm^3^; and (vi) pain on a 0–10 VAS scale during sonopalpation.

It is worth noting that all post-intervention outcomes will be compared to their baseline values at month 0 in order to evaluate their net improvements.

### Sample size

The sample size was calculated with the online calculator “sealed envelope” (www.sealedenvelope.com). Two hundred thirty-two patients are required to have a 95% chance of detecting, with a significant level of 5%, a pain improvement difference of 10% between groups, considering a standard deviation of 10%, a success rate of the rehabilitation of 75% (i.e., for 4 patients included, one is expected to be randomized), and a dropout rate of 10%. The chosen pain improvement was expected to be of 50% into the control group and 60% into the experimental group. The standard deviation of 10% was estimated under the basis of a previous study of Mishra et al. [16]. Because the estimated standard deviation is not fully reliable and the calculated sample size is relatively small, the standard deviation will be re-evaluated and the sample size corrected accordingly if necessary to avoid a potential lack of power (*β*). It will be performed once the primary outcome is available for 40 patients.

### Statistical analysis

The difference in primary outcome (pain improvement from baseline to 3 months post-intervention) between the treatment and control groups will be evaluated using the unpaired Student *T* test (or the Wilcoxon rank test when appropriate). The difference in secondary outcomes (changes from baseline to other time points) between the treatment and control groups will be evaluated with the appropriate statistical test (categorical variables: chi-squared, Fisher’s exact; continuous variables: Student’s or Wilcoxon rank tests). All analyses will be performed with an intention-to-treat analysis. Estimates of effect, 95% confidence intervals and descriptive *p* values will be reported whenever possible. In addition, graphs will be presented whenever possible.

Because the intervention is not considered at risk, not interim analysis is planned. However, in order to re-adjust the sample size calculation, it is planned to re-assess the standard deviation of the primary outcome for the 40 first allocated patients.

In case of missing data on the primary outcome, patients will be withdrawn. Patients with missing data for any of the secondary outcomes will be kept on the study, but excluded from the corresponding analysis.

### Data collection and management

All data collected in this trial will be recorded on standardized paper case report forms (CRF). The sponsor-investigator (AS) is responsible for ensuring that all parts of the CRFs are filled in correctly. The two used questionnaires, SANE score [22] and the PRTEE score [25], are commonly used validated tools. The principal investigator is responsible for ensuring that all parts of CRFs are filled in correctly. Any change or correction to the CRF should be dated and initialed. Each CRF must be signed at least once by the investigator.

The physician closely supervises the participants during rehabilitation visits in order to improve their compliance to the rehabilitation program (CF above). In case of missed appointment, a new date will be proposed by phone. If participants deviate from protocol, the remaining follow-up visits will still be performed and the corresponding data will be collected.

All protocol-required information collected during the trial must be entered by the sponsor-investigator, or designated co-investigator, in the CRF. The CRF pages should be completed and signed the same day that a trial subject is seen for any of the trial procedures. For all CRFs, a copy is immediately stored on the digital secured server of the principal investigator. In order to ensure that the database reproduces the CRFs correctly, the sponsor-investigator, or designated representative, will perform a double entry of the data on two distinct CSV files. For each visit, a different CSV file will be created. Then, the CSV files will be compiled on a database using “R” software (R Foundation for Statistical Computing, Vienna, Austria). The quality of the data entered is guaranteed using “R” software as follows: (i) the duplicate CSV for double entry are digitally compared, and discordant results identified, and (ii) the completeness, validity, and plausibility of the data will be tested for each variable. In case of discordant data, the principal investigator will clarify or correct the problematic data. All eventual changes will be recorded. If no further corrections have to be made in the database, the latter will be closed and used for statistical analyses. All important trial documents (e.g., CRFs) will be archived for at least 10 years after completion of the clinical trial.

Trial data of the patient will be stored in a coded manner. The names of the patients will not be disclosed on CRF. A sequential unique patient number will be attributed to each patient into the trial and reported on the CRF. Identification of patients must be guaranteed at the center. Identification of the patients will be stored on a sequential list stored in the principal investigator’s secured server. The principal investigator and designated representatives will have access to the coded dataset and the identification list during, at the end, and after the study.

Note: in order to insure the blinding of the principal investigator, the allocation list will only be accessible to the co-investigator (MF), or under request to the informatics crew for backup.

### Oversight and monitoring

Prof. Charles Benaïm (CB), chief physician of the Physical Medicine & Rehabilitation Department of the Lausanne University Hospital, will monitor this study, as follows: (i) At the beginning of the study, he will ensure that the randomization and blinding procedure will be respected, first with a sham patient. He will then monitor the first injection procedure for the first patient and ensure that the concealment of allocation is respected. (ii) The first 3 months, then every 3 months, he will ensure that consent forms and CRF are correctly fulfilled and stored. He therefore also ensures that all data are available for the final data analysis. (iii) All kinds of adverse events must be transmitted to him. He will manage or supervise reporting of adverse events and follow-up of concerned patients. (iv) He will be advised of all withdrawals or discontinuations of patients. (v) He will verify the reliability of the statistical analysis. (vi) If deemed necessary, he will plan additional monitoring visits for either the intervention or the data collection.

Adverse events and serious adverse events are recorded in the CRF. Serious adverse events are recorded in dedicated forms and reported to the ethical committee within 7 days. Adverse reactions or suspected unexpected serious adverse reactions are recorded in dedicated forms and reported to the ethical committee within 7 days.

Regular audits are not intended. For the purpose of onsite inspection or audit, the competent authorities or ethics committee may require access to all source documents, CRF, and other trial-related records. The principal investigator must ensure availability of these documents and support the work at any time.

## Discussion

The presented study design allows the investigators to assess the usefulness of PRP as adjuvant therapy to ECRB needling in case of tennis elbow with a randomized controlled trial and to evaluate the effectiveness of a comprehensive integrative rehabilitation protocol. The main indication for infiltrative therapy for epicondylitis is the failure of the first-line treatment. To the best of our knowledge, the most efficient infiltrative procedure consists of tendon fenestration. Whether PRP should be added or not during the tendon fenestration remains debated. There are virtually no contraindications for tendon fenestration and PRP in case of epicondylitis.

In the most recent meta-analysis focused on the management of tendinopathies, PRP was reported to be beneficial compared to others infiltrative therapies [12, 25, 26]. Many of the studies included comparisons between PRP and corticosteroids [7, 27-30]. Given that corticosteroid infiltrations have been shown to be deleterious for epicondylitis [5], the authors considered that the potential observed PRP benefits reported in recent meta-analyses including corticosteroids in control group [12, 25, 26] should not support the use of the PRP itself in clinical practice. Rather, the efficacy of PRP in addition to tendon fenestration compared to tendon fenestration alone remains controversial. Martin et al. [14] found in a partially blinded randomized controlled trial (RCT) involving 71 patients no clinical differences at 6 months of follow-up between 2 sessions of fenestration with either saline + local anesthetic or PRP + local anesthetic. In a similar blinded RCT involving 50 patients, Schöffl et al. [15] found no clinical differences at 6 months of follow-up. Montalvan et al. [16] found in an RCT involving 50 patients no clinical differences at 6 months of follow-up between 2 infiltrations of PRP and saline solutions. Rehabilitation was not allowed during the trial and the tendon was not fenestrated. Mishra et al. [17] reported in a blinded RCT involving 119 patients a positive clinical effect of PRP over saline solution, using a single injection with fenestration. Behera et al. [18] found similar results in a small RCT on 25 patients.

The main strength of the present study design is the case series preceding the RCT. First, it allows to study the effectiveness of combined rehabilitative therapies on symptoms relapse, as well as to standardize the studied population for the RCT. Second and most important, the studied population for the RCT will be better standardized (i.e., all patients with epicondylitis refractory to the same first-line therapies program). The reproducibility of the results will therefore be strengthened. Finally, publishing both the case series and the RCT together will help establishing a comprehensive complete management of the pathology, and giving clinical recommendations in case of positive outcomes. On the other hand, in case of suboptimal clinical outcomes, the presented study design could be reworked in a future trial.

The first main limitation is the small minimum sample size for the RCT (58 patients), chosen to detect a pain improvement difference of 10% between groups. Indeed, in case of success of the rehabilitation protocol, not more than 58 patients will be randomized. It could even be necessary to increase the initial sample size of 232 patients during the study. This small sample size of 58 patients might be prone to sampling variability, reason why stratification is planned. Authors consider that detecting a difference between groups smaller than 10% is not relevant, because the use of PRP should be supported by differences that are clinically relevant rather than statistically significant. PRP is considered as a safe treatment, widely used in sports medicine and promoted by medical companies. Financial interest might therefore easily influence the therapeutic decisions, even if the PRP cost-effectiveness has never been clearly demonstrated for tendon use [26–28]. The second main limitation concerns the inherent variability of the rehabilitation protocol. Indeed, like in virtually all case series with a rehabilitation protocol that include different items, the patient’s implication can differ. Moreover, it is difficult to apply the ESWT therapy with a strict standardized protocol for different reasons. First, because the benefits of ESWT treatment can be achieved after a variable amount of time, the rehabilitation length before the intervention varies between 3 and 6 months. Second, the tolerability and energy delivered can differ between patients. Finally, because the rehabilitation protocol including ESWT is applied to all patients, it is not possible to differentiate the effect of ESWT from other items of the rehabilitation.

The results of this study will provide insights into the effect of PRP as adjuvant therapy to tendon fenestration and may help identifying the best preceding and concomitant rehabilitation protocol.

## Trial status

The trial is currently running. The protocol used is the 2nd version submitted to the ethical committee board (October 3, 2019). The recruitment started in December 2019 and is planned to be completed in December 2023.

## Supplementary information

**Additional file 1.** Rehabilitation protocol

## Data Availability

Data sharing is not applicable to this article as no datasets were generated or analyzed during the current study. This study was approved by the Lausanne ethical committee, Switzerland (CER-VD; ID 2019-01621). All patients must have signed the informed consent form.

## Abbreviations

CRF: Case report forms
ECRB: Extensor carpalis radialis brevis
ESWT: Extracorporal shock waves therapy
PRP: Platelet-rich plasma
PRTEE: Patient-Rated Tennis Elbow Evaluation
RCT: Randomized controlled trial
SANE: Single Assessment Numeric Evaluation
VAS: Visual Analog Scale
