# Supplementary material for: Tennis elbow, study protocol for a randomized clinical trial: needling with and without platelet-rich plasma after failure of up-to-date rehabilitation
Source: J Orthop Surg Res. 2020 Oct 7;15:462. doi: 10.1186/s13018-020-01998-8 (PMC7542691; doi:10.1186/s13018-020-01998-8)
Supplement: Supplementary file 1 — Additional file 1. Rehabilitation protocol [file 13018_2020_1998_MOESM1_ESM.docx]

**REHABILITATION PROTOCOL**

**Study « Tennis Elbow, study protocol for a randomized clinical trial: Needling With and Without Platelet-rich Plasma After Failure of Up-to-date Rehabilitation »**

**Clinicaltrials:** https://clinicaltrials.gov/ct2/show/record/NCT03987256

| **Semaine** | 1 | | 2 | | 3 | | 4 | | 5 | | 6 | | 7 | | 8 | | 9 | | 10 | | 11 | | 12 | |
| --- | --- | --- | --- | --- | --- | --- | --- | --- | --- | --- | --- | --- | --- | --- | --- | --- | --- | --- | --- | --- | --- | --- | --- | --- |
| Ergonomic advice | x |  | x |  | x |  | x |  |  |  |  |  | X |  |  |  |  |  | X |  |  |  |  |  |
| Deep massages | x | x | x | x | x | x | x | x |  |  |  |  |  |  |  |  |  |  |  |  |  |  |  |  |
| Self-exercises | X | X | X | X | X | X | X | X | X | X | X | X | X | (X) | X | (X) | X | (X) | X | (X) | X | (X) | X | (X) |
| Trigger points | X | X | X | X | X | X | X | X | X | X | X | X | X | (X) | X | (X) | X | (X) | X | (X) | X | (X) | X | (X) |
| Manual therapies | (X) | (X) | (X) | (X) | (X) | (X) | (X) | (X) | (X) | (X) | (X) | (X) | (X) | (X) | (X) | (X) | (X) | (X) | (X) | (X) | (X) | (X) | (X) | (X) |
| Kinesiotaping | X | X | X | X | X | X | X | X | (X) | (X) | (X) | (X) | (X) | (X) | (X) | (X) | (X) | (X) | (X) | (X) | (X) | (X) | (X) | (X) |
| Shock waves |  |  |  |  |  |  |  |  | x |  | x |  | x |  | x |  | (x) |  | (x) |  | (x) |  | (x) |  |
| Prescription post. holder | (x) |  |  |  |  |  |  |  |  |  |  |  |  |  |  |  |  |  |  |  |  |  |  |  |
| Prescription orthèse |  |  |  |  |  |  |  |  |  |  |  |  | (X) |  |  |  |  |  |  |  |  |  |  |  |

X = Implementation in all cases

(X) = Implementation according to the therapist's judgment

**Ergonomic advice, adaptation of activities (physio and doctor)**

1. *1. Professional (including desk adaptation, eg "vertical" mouse, desk height).*
2. *2. Sports (improved gesture, "tennis racket handle" equipment, frequency and intensity of activity adaptation*).
3. *Daily life.*

**Deep massages**

Between 10 and 15 minutes of transverse friction / sliding / deep massage during a 30-minute therapy, seeking to find the trigger point (point of intense pain) in the forearm, wrist, scapula and scalene. The force to be applied is on a scale between 5 and 8 VAS.

***
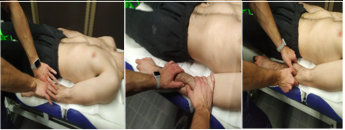
***

**Work on trigger points**

1. Research and treatment of trigger points (epicondylar and associated muscle chains)
2. manual :

**
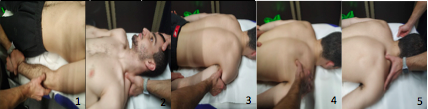
**

1. dry needling if refractory to 2 weeks of manual treatment.


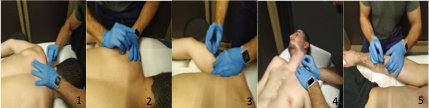


**Manual therapies**

1. Mobilization according to Mulligan in the event of frank hypomobility (fascia, muscle, joint).


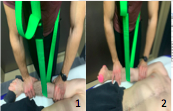


1. Postural reconstruction in the event of a clear postural problem participating in pain prescription of a "posture holder" if necessary.


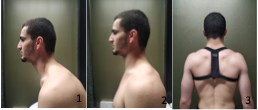


*Good posture includes: aligning your back, shoulders (back and bottom) and your head in line with your shoulders with your eyes on the horizon. Principle to apply standing and sitting.*

1. Postural and proprioceptive self-exercises with Foam Roll Triger Point Massage Ball.

| ***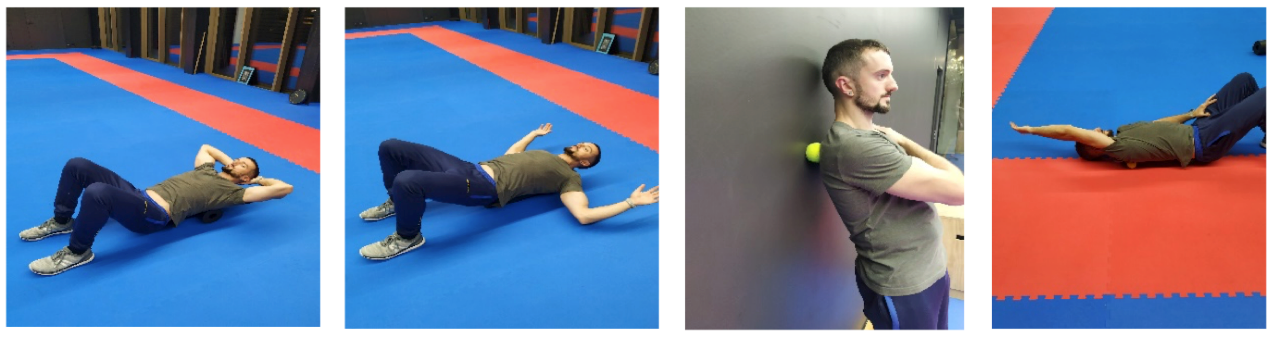*** |
| --- |

**Strengthening and stretching**

Explanatory text of the different stages of reinforcement.

1. Progressive enhancement of eccentric strengthening 7/7.

| 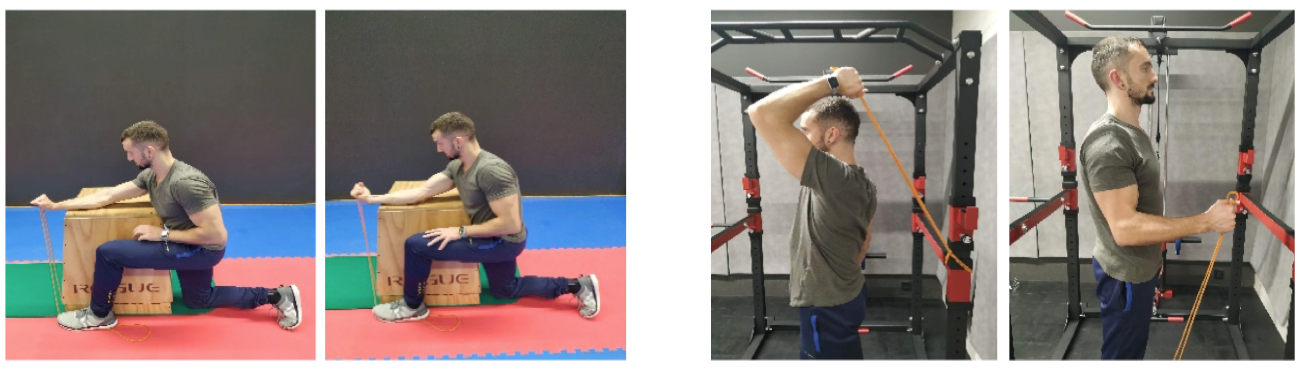   1. 1st stage (B) 2nd stage |
| --- |


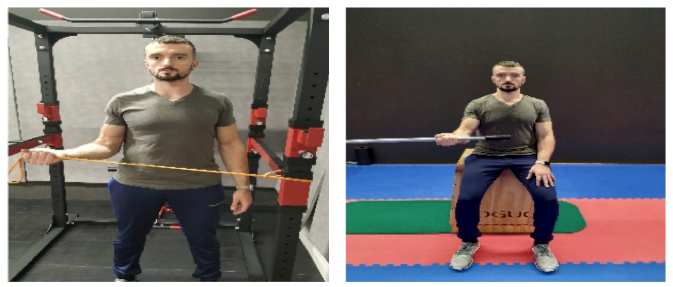


(C) 3rd stage


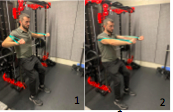


- Concentric and eccentric reinforcement with rear elastic band, moderate pain (3/10) tolerated.


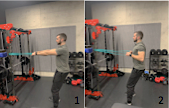


- Concentric and eccentric reinforcement with elastic band in front, moderate pain (3/10) tolerated.

1. Stretching of the muscular chains in the event of stiffness.

| 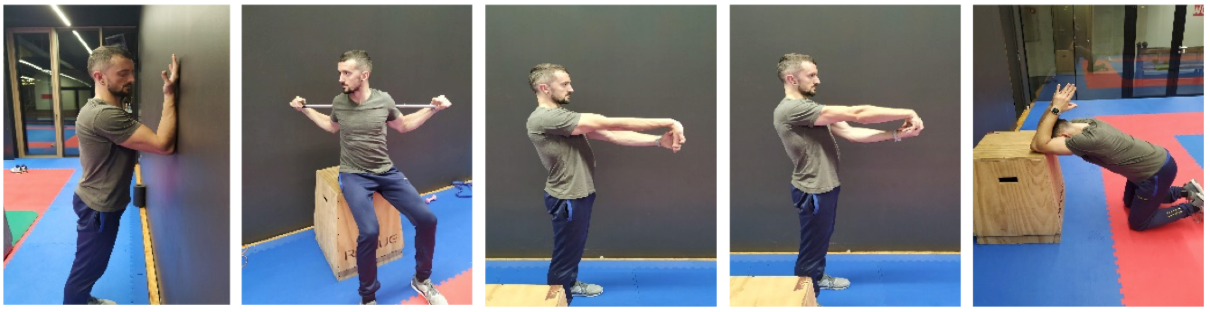 |
| --- |

1. Neurodynamic stretching if the tension is radicular.


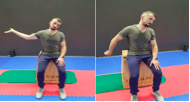


1. Overall strengthening of the upper limb, including periscapular 7/7.

| 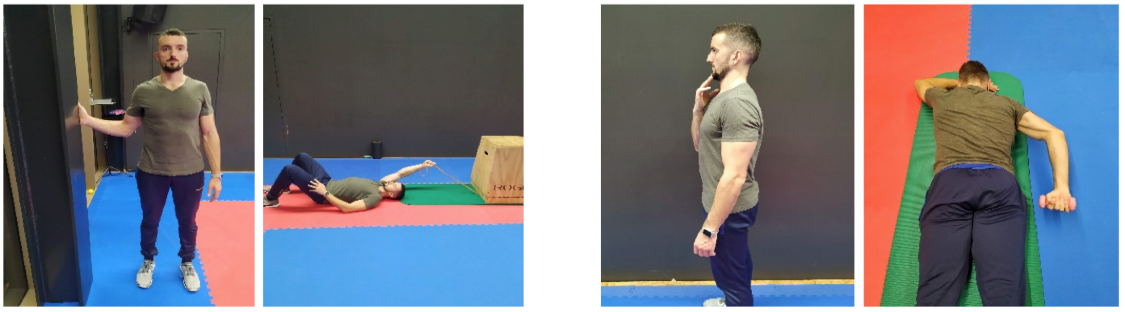  1^st^ Stage 2^nd^ Stage |
| --- |
| 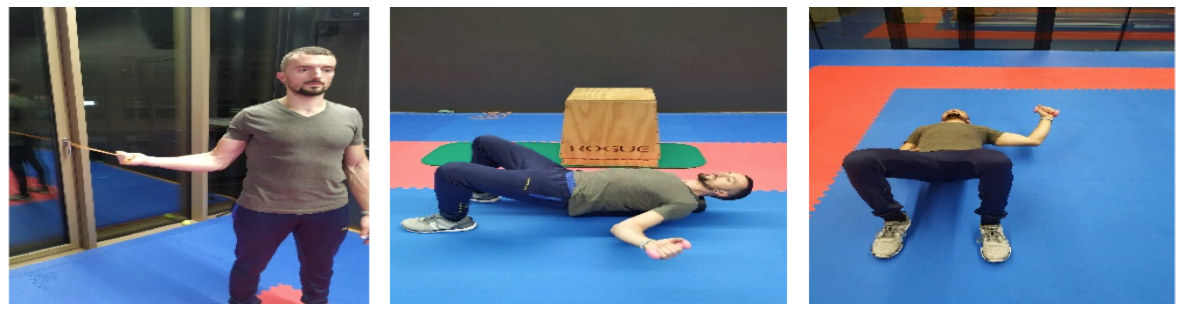  3 ^rd^ stage |

**Focused shock waves**

4-8 sessions, 0.15 - 0.35 mj / mm2 depending of the tolerance, 2000 pulses, 4-5 Hz.

**Kinesiotaping**

Taping application.


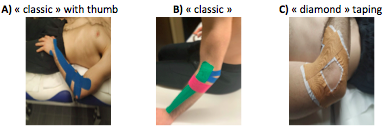


1. Tension of 10-15% on long application and 50% on perpendicular application (elastic tape).
2. Tension of 10-15% on long application and 50% on perpendicular application (non elastic tape).
3. Taping rigide (non élastique).

**Orthosis (Epicondylitis Brace)**

Used at 6 weeks if taping deemed insufficiently effective to protect from pain during activities with no force or with minimal force.

**REFERENCES**

1. Loew LM, Brosseau L, Tugwell P, Wells GA, Welch V, Shea B, et al. Deep transverse friction massage for treating lateral elbow or lateral knee tendinitis. Cochrane Database Syst Rev. 2014(11):CD003528.

2. Pitsillides A, Stasinopoulos D. Cyriax Friction Massage-Suggestions for Improvements. Medicina (Kaunas, Lithuania). 2019;55(5).

3. Hoogvliet P, Randsdorp MS, Dingemanse R, Koes BW, Huisstede BM. Does effectiveness of exercise therapy and mobilisation techniques offer guidance for the treatment of lateral and medial epicondylitis? A systematic review. Br J Sports Med. 2013;47(17):1112-9.

4. Pecos-Martin D, Ponce-Castro MJ, Jimenez-Rejano JJ, Nunez-Nagy S, Calvo-Lobo C, Gallego-Izquierdo T. Immediate effects of variable durations of pressure release technique on latent myofascial trigger points of the levator scapulae: a double-blinded randomised clinical trial. Acupunct Med. 2019;37(3):141-50.

5. Ransone JW, Schmidt J, Crawford SK, Walker J. Effect of manual compressive therapy on latent myofascial trigger point pressure pain thresholds. J Bodyw Mov Ther. 2019;23(4):792-8.

6. Espejo-Antunez L, Tejeda JF, Albornoz-Cabello M, Rodriguez-Mansilla J, de la Cruz-Torres B, Ribeiro F, et al. Dry needling in the management of myofascial trigger points: A systematic review of randomized controlled trials. Complement Ther Med. 2017;33:46-57.

7. Liu L, Huang QM, Liu QG, Ye G, Bo CZ, Chen MJ, et al. Effectiveness of dry needling for myofascial trigger points associated with neck and shoulder pain: a systematic review and meta-analysis. Archives of physical medicine and rehabilitation. 2015;96(5):944-55.

8. Reyhan AC, Sindel D, Dereli EE. The effects of Mulligan's mobilization with movement technique in patients with lateral epicondylitis. J Back Musculoskelet Rehabil. 2019.

9. Arshadi R, Ghasemi GA, Samadi H. Effects of an 8-week selective corrective exercises program on electromyography activity of scapular and neck muscles in persons with upper crossed syndrome: Randomized controlled trial. Phys Ther Sport. 2019;37:113-9.

10. Pienimaki T, Karinen P, Kemila T, Koivukangas P, Vanharanta H. Long-term follow-up of conservatively treated chronic tennis elbow patients. A prospective and retrospective analysis. Scand J Rehabil Med. 1998;30(3):159-66.

11. Smidt N, Lewis M, DA VDW, Hay EM, Bouter LM, Croft P. Lateral epicondylitis in general practice: course and prognostic indicators of outcome. J Rheumatol. 2006;33(10):2053-59.

12. Croisier JL, Foidart-Dessalle M, Tinant F, Crielaard JM, Forthomme B. An isokinetic eccentric programme for the management of chronic lateral epicondylar tendinopathy. Br J Sports Med. 2007;41(4):269-75.

13. Cook JL, Rio E, Purdam CR, Docking SI. Revisiting the continuum model of tendon pathology: what is its merit in clinical practice and research? Br J Sports Med. 2016;50(19):1187-91.

14. Khan KM, Cook JL, Kannus P, Maffulli N, Bonar SF. Time to abandon the "tendinitis" myth. BMJ. 2002;324(7338):626-7.

15. Scott A, Squier K, Alfredson H, Bahr R, Cook JL, Coombes B, et al. ICON 2019: International Scientific Tendinopathy Symposium Consensus: Clinical Terminology. Br J Sports Med. 2019.

16. Voleti PB, Buckley MR, Soslowsky LJ. Tendon healing: repair and regeneration. Annu Rev Biomed Eng. 2012;14:47-71.

17. Freitas SR, Vaz JR, Bruno PM, Andrade R, Mil-Homens P. Stretching Effects: High-intensity & Moderate-duration vs. Low-intensity & Long-duration. Int J Sports Med. 2016;37(3):239-44.

18. De-la-Llave-Rincon AI, Ortega-Santiago R, Ambite-Quesada S, Gil-Crujera A, Puentedura EJ, Valenza MC, et al. Response of pain intensity to soft tissue mobilization and neurodynamic technique: a series of 18 patients with chronic carpal tunnel syndrome. J Manipulative Physiol Ther. 2012;35(6):420-7.

19. Sethi K, Noohu MM. Scapular muscles strengthening on pain, functional outcome and muscle activity in chronic lateral epicondylalgia. J Orthop Sci. 2018;23(5):777-82.

20. Thiele S, Thiele R, Gerdesmeyer L. Lateral epicondylitis: This is still a main indication for extracorporeal shockwave therapy. Int J Surg. 2015;24(Pt B):165-70.

21. Goel R, Balthilaya G, Reddy RSY. EFFECT OF KINESIO TAPING VERSUS ATHLETIC TAPING ON PAIN AND MUSCLE PERFORMANCE IN LATERAL EPICONDYLALGIA. International Journal of Physiotherapy and Research. 2015;3:839-44.

22. Eraslan L, Yuce D, Erbilici A, Baltaci G. Does Kinesiotaping improve pain and functionality in patients with newly diagnosed lateral epicondylitis? Knee surgery, sports traumatology, arthroscopy : official journal of the ESSKA. 2018;26(3):938-45.

23. Giray E, Karali-Bingul D, Akyuz G. The Effectiveness of Kinesiotaping, Sham Taping or Exercises Only in Lateral Epicondylitis Treatment: A Randomized Controlled Study. PM R. 2019;11(7):681-93.

24. Dilek B, Batmaz I, Sariyildiz MA, Sahin E, Ilter L, Gulbahar S, et al. Kinesio taping in patients with lateral epicondylitis. J Back Musculoskelet Rehabil. 2016;29(4):853-8.

25. Struijs PA, Kerkhoffs GM, Assendelft WJ, Van Dijk CN. Conservative treatment of lateral epicondylitis: brace versus physical therapy or a combination of both-a randomized clinical trial. The American journal of sports medicine. 2004;32(2):462-9.
